# Crop sensitivity to waterlogging mediated by soil temperature and growth stage

**DOI:** 10.3389/fpls.2023.1262001

**Published:** 2023-10-26

**Authors:** Fu-Li Xu, Pei-Min Hu, Xiao Wan, Matthew Tom Harrison, Ke Liu, Qin-Xue Xiong

**Affiliations:** ^1^ College of Agriculture, Yangtze University, Jingzhou, China; ^2^ Meteorological Service Center, Jingzhou Meteorological Bureau, Jingzhou, China; ^3^ Tasmanian Institute of Agriculture, University of Tasmania, Launceston, TAS, Australia

**Keywords:** waterlogging, SWAGMAN Destiny, hypoxia, anoxia, abiotic stress, soil aeration, crop physiology, model

## Abstract

Waterlogging constrains crop yields in many regions around the world. Despite this, key drivers of crop sensitivity to waterlogging have received little attention. Here, we compare the ability of the SWAGMAN Destiny and CERES models in simulating soil aeration index, a variable contemporaneously used to compute three distinct waterlogging indices, denoted hereafter as WI _Destiny_, WI_ASD1_, and WI_ASD2_. We then account for effects of crop growth stage and soil temperature on waterlogging impact by introducing waterlogging severity indices, WI _Growth_, which accommodates growth stage tolerance, and WI _Plus_, which accounts for both soil temperature and growth stage. We evaluate these indices using data collected in pot experiments with genotypes “Yang mai 11” and “Zheng mai 7698” that were exposed to both single and double waterlogging events. We found that WI _Plus_ exhibited the highest correlation with yield (-0.82 to -0.86) suggesting that waterlogging indices which integrate effects of temperature and growth stage may improve projections of yield penalty elicited by waterlogging. Importantly, WI _Plus_ not only allows insight into physiological determinants, but also lends itself to remote computation through satellite imagery. As such, this index holds promise in scalable monitoring and forecasting of crop waterlogging.

## 1 Introduction

In theory, optimal plant water balance may be realized through harmony between root water absorption and leaf transpiration (Ibrahim et al., 2018). Adequate water supply is essential for healthy plant growth, but when the soil becomes saturated, potentially resulting in plant waterlogging stress (Singhal et al., 2022). Soil waterlogging can manifest in agricultural fields for various reasons, such as excessive rainfall or irrigation, inadequate soil drainage, rising or perched water tables and lateral surface or subsurface flows (Liu et al., 2021). This can deplete oxygen levels within soil pores, leading to diminished growth, senescence and, in severe cases, crop mortality (Githui et al., 2022). Waterlogging can also indirectly impact on growth via soil mineral nitrogen, organic matter and carbon, as well as soil microbiota (Phelan et al., 2015; Rawnsley et al., 2019; Sándor et al., 2020).

The middle and lower reaches of the Yangtze River region are among China’s primary wheat production zones, but they are also severely afflicted by waterlogging (Wan et al., 2022). Around 41% of total arable land area in this region grapples with waterlogging, significantly hampering the consistency and stability of crop yields (Hu, 2023). Investigating the impact of waterlogging, particularly hypoxia stress, on wheat growth and yield is crucial, together with how waterlogging may be amplified or interplay with other crop stresses, such as extreme heat, that may occur later in the growing season (Langworthy et al., 2018; Harrison, 2021).

Two approaches are typically employed to assess the extent of crop waterlogging. The first relies on soil groundwater depth, often computed in crop models using algorithms pertaining to cumulative excess groundwater depth (SEW30) (Kanwar et al., 1988; Qian et al., 2015) and consecutive suppression days of waterlogging (CSDI) (Evans and Skaggs, 1993). This method primarily elucidates the impact of groundwater and surface water on crop waterlogging stress, yet does not allow other waterlogging pathways to be captured, such as waterlogging triggered by excessive irrigation (Liu et al., 2020). Conversely, the second category is grounded upon occurrence of climatic elements, which then allows computation of indices such as the waterlogged day index (Guo et al., 2016) and wetland day index (Zhang et al., 2019). Such parameters ignore the multifaceted and integrated effects of soil type, topography and hydrology. Although remote sensing may potentially furnish insights into such factors at the regional scale (Shahpari et al., 2021), their availability to practitioners at present is limited.

The period from March to April in the middle and lower reaches of the Yangtze River region typically witnesses total precipitation from 300 to 400 mm, constituting 30% to 40% of the annual rainfall. This timeframe coincides with critical nutritional and reproductive growth stages of wheat crops, and is often punctuated by frequent flood events (Gao et al., 2020). Although wheat has three distinct waterlogging stress responses (tolerant, inhibitory, and adaptive phases) (Shaw and Meyer, 2015), recurring episodes of waterlogging stress can significantly inhibit growth and yields, especially when high-intensity waterlogging coincides with extreme weather fluctuations. This explains why many models perform well with waterlogging stress under experimental conditions, but fall short of accurately predicting outcomes under field conditions (Shaw et al., 2013; Harrison et al., 2019).

Broadly, two approaches are employed to simulate waterlogging in crop models. The first calculates the Stress Day Index (SDI) based on groundwater table depth, as exemplified by DRAINMOD (Skaggs, 2018). However, this approach overlooks genotypic differences in crops, considering only groundwater and surface water, rendering it unsuitable for addressing waterlogging stemming from overirrigation. The second method computes hypoxic stress as a function of soil moisture. Notable examples include CROPR (Qian et al., 2017), SWAGMAN Destiny (Meyer et al., 1996), and the Agricultural Production Systems Simulator (APSIM) (Asseng et al., 1998; Liu et al., 2021). Shaw and Meyer (2015) showed that yield reductions were 6% higher at 65% air-filled pore space compared to 10%, leading to their proposal of three stages for simulating plant responses to waterlogging damage. Their hypoxic stress factor accounts for waterlogging duration and crop tolerance, making it applicable to damage caused by excessive irrigation and facilitating more precise quantitative damage analysis (Shaw et al., 2013). The Crop Estimation through Resource and Environment Synthesis (CERES) model, commonly used for predicting crop growth and yield, has been enhanced by Lizaso et al., who successfully integrated low oxygen (anaerobic) stress factors into the CERES-Wheat model (Lizaso and Ritchie, 1997).

The objectives of this study were thus to quantify waterlogging by integrating computations from both the SWAGMAN Destiny and CERES models. Specifically, we develop five wheat waterlogging injured indices: WI_ASD2_ and WI_ASD1_ were conceptualized as adaptations of the CERES model (Lizaso and Ritchie, 1997), WI _Destiny_ was derived from the SWAGMAN Destiny model, WI _Growth_ introduces differences in waterlogging tolerance across crop growth stages (following WI _Destiny_), while WI _Plus_ considers the influence of soil temperature in addition to WI _Growth_. To assess the accuracy of these indicators, we conducted pot experiments with winter wheat (involving single or double flooding) using local genotypes (“Yang mai 11” and “Zheng mai 7698”). By analyzing relationships between our WIs and yield, we provide insight into how integrated physiological determinants impact on crop yield.

## 2 Material and methods

### 2.1 Plant material and experimental design

The study region is located in the Jianghan Plain, Hubei Province, China, characterized by a subtropical monsoon climate. The region’s primary crops include rice, wheat, rapeseed, and cotton. Two wheat varieties were employed in the experiments: the waterlog-resistant Yang mai 11 (referred to as Y) and the waterlog-unresistant Zheng mai 7698 (referred to as Z) (Yu et al., 2014). The soil used in the pot experiment was selected from paddy soil, which is a typical cultivated soil in the middle and lower reaches of the Yangtze River (Gao et al., 2020).

The winter wheat pot experiment with waterlogging spanned from November 2020 to May 2022 and was conducted at the open field experimental site of the College of Agriculture, Yangtze University (longitude 112°08’, latitude 30°21’). This two-year experiment utilized storage boxes measuring 60 cm in length, 45 cm in width, and 35 cm in height, arranged in a randomized complete block design.

The soil’s basic physicochemical properties were as follows: pH value of 7.85, total nitrogen content of 1.19 g/kg, total phosphorus content of 0.77 g/kg, total potassium content of 10.45 g/kg, organic matter content of 15.84 g/kg, alkaline nitrogen content of 58.44 mg/kg, effective phosphorus content of 31.12 mg/kg, and available potassium content of 106.09 mg/kg. Wheat was sown on November 3, 2020, with each box receiving a mixture of 17.78g/kg of compound fertilizer (N: P_2_O_5_:K_2_O=15:15:15), 0.89g/kg of KCl, and 3.94g/kg of urea before sowing. At the tillering stage on January 25, 2022, urea was applied at a rate of 4.17 g/kg.

During the experiment, single waterlogging treatments lasting for 5 days, 12 days, and 20 days were administered on March 21 and April 8 in 2021 (or 2022). Following a 10-day interval, a second round of waterlogging treatments for the same durations (i.e., double waterlogging treatments) was applied to the potted plants in multiple boxes. Waterlogging was manually induced by irrigating with marked buckets to maintain soil moisture content at over 90% of the maximum field water-holding capacity. The criterion for waterlogging stress was maintaining a water level 5 centimeters above the soil surface. Daily irrigation volumes (in liters) for each box were meticulously recorded.

The experiment adopted a randomized block design involving continuous irrigation treatments during two critical periods: from tillering to flowering (March 21st to April 7th, denoted as B) and from flowering to maturity (April 8th to May 8th, denoted as Y), which corresponds to the crucial water requirement period for wheat. The experiment included three different durations of irrigation (5 days, 12 days, 20 days) and two types of irrigation treatments (single continuous waterlogging and double continuous waterlogging), resulting in a total of 72 treatments. Each treatment was replicated three times, with an additional six control boxes. Protective rows were established around the experiment using boxes without irrigation, featuring either the “Yang mai 11” variety or the “Zheng mai 7698” variety. The control boxes were positioned at the center. In total, the experiment encompassed 120 boxes.

### 2.2 Measurement indexes and methods

Soil Moisture: soil volumetric water content was assessed using an EM50 soil moisture measurement device connected to an EC-5 soil moisture sensor. The water content probe was inserted into the soil at a depth of 5-6cm. Observations were conducted daily from 8:00 to 20:00, with measurements taken at hourly intervals. The daily soil volumetric moisture content was calculated as the average of the 24-hour observation period.

Soil Temperature: Temperature data recorded using a fiber optic oxygen meter (PreSens Microx4, Germany Regensburg) with the measuring part inserted 5-6 cm into the soil. Then take the average as the daily average soil temperature.

Yield Measurement: At maturity (May 8), winter wheat from all boxes was harvested. The harvested wheat was threshed, sun-dried, and subsequently winnowed to remove impurities and empty grains. The total weight of the dried grains was then measured.

Meteorological Elements: The experimental station was equipped with a HOBO automatic weather station (HOBO Micro-Weather Station) that automatically recorded daily rainfall and other meteorological data.

### 2.3 Calculation method of wheat waterlogging injured index

*WI_Destiny_* takes into account the combined effects of waterlogging duration and soil moisture on the root system. *WI_ASD_*_1_ and *WI_ASD_*_2_ are improvements made by Lizaso, J. I. et al. to the CERES model, where the growth stage is divided into 1-9 phases (Lizaso and Ritchie, 1997). However, this method has only been validated in research on the impact of waterlogging on maize growth and development, and there is no relevant verification for wheat.

Crop sensitivity to waterlogging stress is heavily dependent on the developmental stage at which waterlogging occurs (Liu et al., 2023). As such, considering the lack of research on waterlogging tolerance during different growth stages in *WI_Destiny_*, we have made modifications by using a sigmoid function to simulate the differences in waterlogging tolerance. This has led us to construct a new index called *WI*_Growth_.

**Figure 1:**
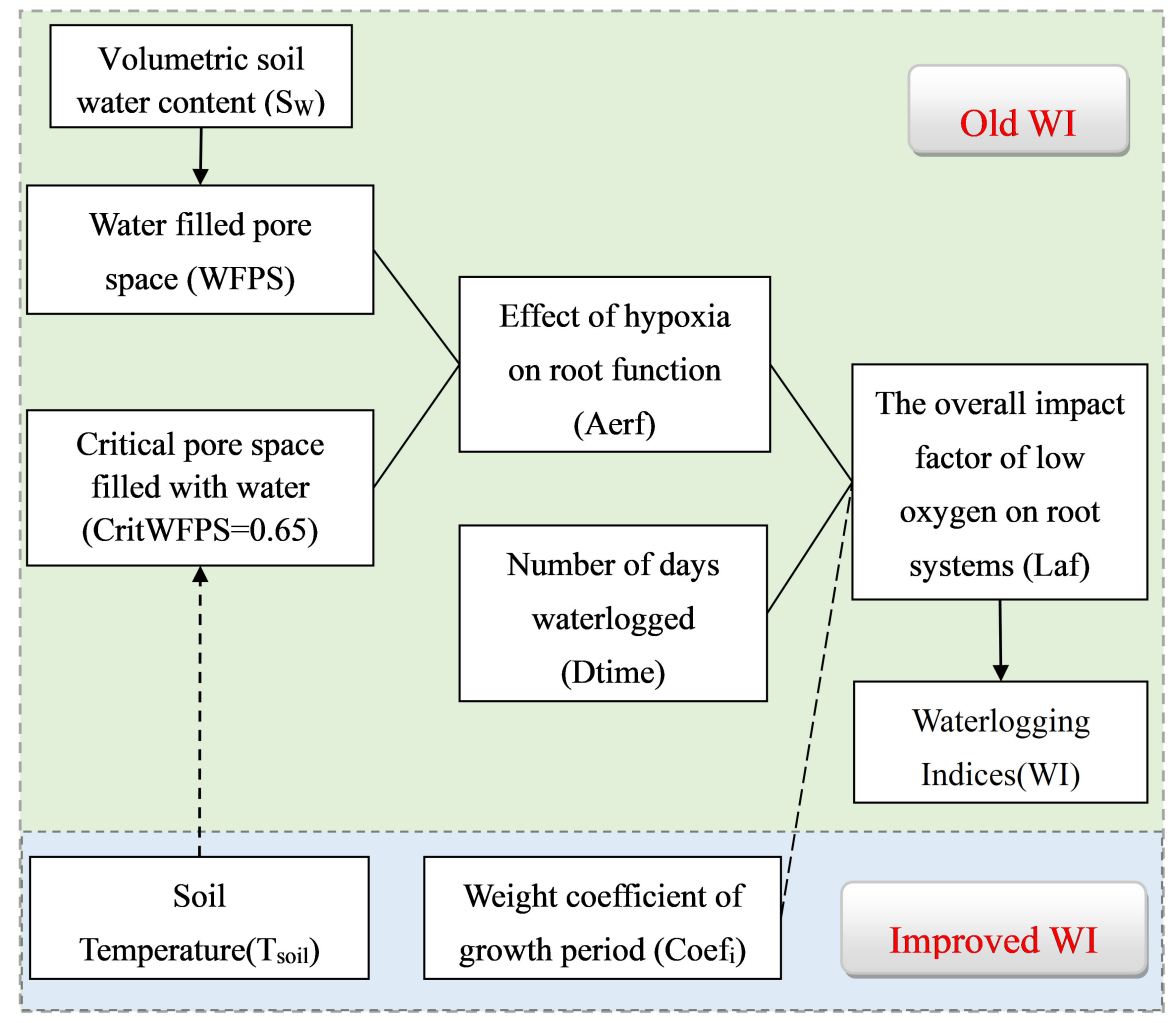
Sequence diagram of the components required to compute WI Destiny in SWAGMAN destiny (Old WI) and its refinement (Improved WI). WI _Growth_ primarily involved the addition of the weight coefficient for the growth period (Coef_i_). WI _Plus_ incorporated the Soil Temperature (T_soil_) and the Weight coefficient of growth period (Coef_i_).

Constant critical values (0.65) have frequently been applied to represent oxygen stress (Barber et al., 2004; Leão et al., 2006), but constants are unlikely to be sufficient for any proxy. In the SWAGMAN Destiny model, the critical values for oxygen stress are likely to be inappropriate and must change with different soil types, varied temperature, organic matter content, soil depth and plant characteristics. Among them, the main factor is soil temperature (Bartholomeus et al., 2008). Therefore, based on *WI*_Growth_, we have incorporated soil temperature and made improvements to the critical water filled pore space (CritWFPS=0.65), resulting in a revised index called *WI_Plus_*. The improvement of the SWAGMAN Destiny model is shown in **Figure 1**.

#### 2.3.1 Calculating waterlogging injured index from SWAGMAN Destiny model (*WI_Destiny_*)

In the SWAGMAN Destiny model, the calculation process for total influence factors of soil aeration index on roots involves several steps (**Figure 1**). Firstly, it is calculated the soil pore water content (*WFPS*) by obtaining the daily water content of the soil surfaces (*S_W_*). Then, the characteristic parameter (*A_erf_*) of the effect of low oxygen on the root system was calculated. Finally, combining the characteristic parameters (*A_erf_*) with the duration of continuous waterlogging (*D_time_*) to calculate the overall impact factor of low oxygen on root systems (*L_af_*), providing a comprehensive assessment of the degree of impact of low oxygen stress on roots. The calculation process is as follows:

(1) Characteristic Parameters of hypoxia’s impact on root function (*A_erf_*)

The first step is to calculate the relative amount of pore space filled with water (Aerf) within the soil. This is a zero to unity factor where zero indicates the soil pores are completely full of water (the soil is saturated) and one indicates there is no water in the soil pores (the soil is oven dry). Aerf is calculated (Shaw et al., 2013) as:


(1) $$A_{erf}=\{\begin{array}{cc} 1-\frac{\left(WFPS-CritWFPS\right)}{\left(1-CritWFPS\right)} & WFPS>CritWFPS\\ 1 & WFPS\leq CritWFPS \end{array}$$

Where


(2) $$WFPS=\frac{S_{W}}{\left(1-\frac{B_{D}}{Soil\;particle\;density}\right)}$$

Where, *WFPS* is water filled pore space at soil water content; *S_W_* is Volumetric soil water content (cm^3^_water_ cm^-3^_soil_); *B_D_* is Bulk density of dry soil (g/cm^-3^), and in accordance with the actual soil bulk density measured in pot experiments, it is taken as 1.4 g·cm^-3^; The general soil particle density falls within the range of 2.6 to 2.8 g/cm^3^, and here it is taken as 2.7 g/cm^3^.

The critical water filled pore space (*CritWFPS*) is set to 0.65. Doran et al. found that respiratory activity of microbial function in soils decreased when the water filled pore space increased to a value above 0.65 (Doran et al., 1990).

(2) Number of days waterlogged (*D_time_*)

When the average daily soil pore water content (*CritWFPS*) is greater than 0.65, it is defined that wheat is affected by waterlogging on that day. If the average daily soil pore water content (*CritWFPS*) is less than 0.65, then wheat is in a normal state with a continuous waterlogging duration of 0 days. Due to the delayed response of the root system to waterlogging damage, it is set that the damage will only affect the crop’s root system after 3 days, and the impact remains unchanged after 60 days (Shaw and Meyer, 2015). The calculation formula is as follows (Hu, 2023):


(3) $$D_{time,i}=\{\begin{array}{cc} 1 & \left(WFPS_{,i}\geq 0.65)\;and\;(WFPS_{,i-1}\geq 0.65\right)\\ and\;(WFPS_{,i-2}\geq 0.65)\\ \;0 & \left(WFPS_{,i}<0.65\right)\\ D_{time,i-1}+1 & \left(D_{time,i-1}\geq 3)\;and\;(WFPS_{,i}\geq 0.65\right)\\ 60 & \left(D_{time,i-1}\geq 60)\;and\;(WFPS_{,i}\geq 0.65\right) \end{array}$$

Where *D_time,i_* represents the duration of waterlogging on the i-th day; *WFPS_,i_* is the WFPS of the i-th day;

(3) Overall impact factor of hypoxia on root function (*L_af_*)

The overall impact function of waterlogging on the root system is a comprehensive consideration of the effects of soil moisture and duration of waterlogging. The *L_af_* value is a characteristic factor ranging from 0 to 1. A value closer to 1 indicates a greater impact of waterlogging on the root system of winter wheat, while a value closer to 0 indicates a lesser impact of waterlogging on the root system of winter wheat. its calculation formula is represented as (Shaw et al., 2013):


(4) $$L_{af,i}={\left(1-Aerf_{i}\right)}^{D_{time,i}{}^{0.167}}$$

(4) Calculation of Waterlogging Index (*WI_Destiny_*).

In the SWAGMAN Destiny model, *L_af_* represents the simulated daily impact of waterlogging on winter wheat. Considering the small magnitude of values, it is multiplied by 1000 for scaling purposes. The daily impact function of wheat is averaged throughout the entire growing season to calculate the Waterlogging Index (*WI_Destiny_*) for the entire crop cycle.


(5) $$WI_{Destiny}=\frac{1}{n}\sum_{i=1}^{n}\left(L_{af,i}\times 1000\right)$$

#### 2.3.2 Calculating waterlogging injured index from CERES model (*WI_ASD_*_1_ and *WI_ASD_*_2_)

Jones et al. proposed a model to predict root growth that includes soil constraints affecting root growth (Jones et al., 1991). The model uses the fraction of water filled pore space as an aeration index to affect, root attributes such as rooting depth, branching and senescence.

(1) Soil aeration index

Aeration indexes are calculated in terms of soil porosity since oxygen diffusion depends on the air-filled pore space. According to formula (2), the water-filled porosity (WFPS) is calculated.

The water-filled pore space at saturation is assumed to have a maximum value (*XWWFPS*) of 0.93 to account for trapped air in the soil profile. Whenever the water-filled pore space (WFPS_i_) in a soil layer is larger than a critical value (*CWFPS*), a layer aeration factor (*LAF_i_*) was calculated as (Lizaso and Ritchie, 1997):


(6) $${\text{LAF}}_{\text{i}}=\left(1-{\text{WFPS}}_{\text{i}}\right)/\left(1-\text{CWFPS}\right)$$

where *CWFPS* is equal to 0.45 and *LAF_i_* cannot be larger than 1. When a layer with *WFPS* of 0.9 or larger limits the aeration of lower layers. The Whole Rhizosphere Aeration Factor (WRAF) for the entire root zone is calculated by integrating all soil layers using root density as the weighting factor. The formula is as follows (Lizaso and Ritchie, 1997):


(7) $$\text{WRAF}=(\sum_{\text{i}=1}^{\text{n}}{\text{LAF}}_{\text{i}}{*\text{RLV}}_{\text{i}})/(\sum_{\text{i}=1}^{\text{n}}{\text{RLV}}_{\text{i}})$$

Where RKV_i_ is the root length density for each soil layer. The soil in the root layer is divided into *n* layers. *WRAF* is a dimensionless unit ranging from 0 to 1, where a value closer to 1 indicates good soil aeration, while a value closer to 0 indicates severe root oxygen stress. Two dimensionless aeration stress indexes, ASD and ASD2, were calculated using WRAF to simulate the cumulative aeration status experienced by the plant.

The two dimensionless aeration stress indexes (ASD1 and ASD2) are calculated using the following formulas (Lizaso and Ritchie, 1997):


(8) $${\text{ASD}1}_{\text{day}}=\{\begin{array}{c} {\text{ASD}1}_{\text{day}-1}+(1-{\text{WRAF}}_{\text{day}})\;\;\;\;\;{\text{ASD}1}_{\text{day}}>{\text{ASD}1}_{\text{day}-1}\\ {\text{ASD}1}_{\text{day}-1}-{\text{WRAF}}_{\text{day}}\times ({\text{WRAF}}_{\text{day}}\times {\text{WRAF}}_{\text{day}-1})\times 0.855\times {\text{ISTAGE}}^{2}\;\;\;\;{\text{ASD}1}_{\text{day}}\leq {\text{ASD}1}_{\text{day}-1} \end{array}$$

${\text{ISTAGE}}^{2}$ is an integer variable (1 to 9) identifying the phenological stage of the crop.


(9) $$\text{ASD}2_{\text{day}}=\{\begin{array}{c} \text{ASD}2_{\text{day}-1}+(1-{\text{WRAF}}_{\text{day}})\;\;\;\;\;\text{ASD}2_{\text{day}}>\text{ASD}2_{\text{day}-1}\\ \text{ASD}2_{\text{day}-1}-{\text{WRAF}}_{\text{day}}\times \text{ASD}2_{\text{day}-1}\times k\;\;\;\;\text{ASD}2_{\text{day}}\leq \text{ASD}2_{\text{day}-1} \end{array}$$

*k* is the coefficient of growth stage. During the vegetative growth stage,*k* =0.8 when ASD2 > 4.0 and *k*=0.1 when ASD2 <4.0. During the remainder of the season, *k*=0.5.

(2) Calculation of $WI_{ASD1}$ and $WI_{ASD2}$ The average ASD value for each day of the entire growth season of winter wheat is calculated to obtain the waterlogging index of the whole reproductive period. The calculation formula is as follows:


(10) $$WI_{ASD1}=\frac{1}{n}\sum_{i=1}^{n}ASD1_{,i}$$

(11) $$WI_{ASD2}=\frac{1}{n}\sum_{i=1}^{n}ASD2_{,i}$$

#### 2.3.3 Calculation of waterlogging injured index for growth stage (*WI*_Growth_)

Modify Equation (4) by introducing the impact function of the growth stage. The calculation process is as follows:


(12) $$G\;Laf_{i}=[{\left(1-Aerf_{i}\right)}^{D_{time,i}{}^{0.167}}]\times Coef_{i}$$

(13) $$WI_{\;\text{Growth}}=\frac{1}{n}\sum_{i=1}^{n}(G\;L_{af,i}\times 1000)$$

Where $Aerf_{i}$ refers to formula (1). $D_{time}$ refers to formula (3). $Coef_{i}$ is the reaction weight coefficient of winter wheat to waterlogging during different growth stages, which ranges from 0 to 1. The impact of winter flooding on winter wheat is generally not significant during the overwintering period, with a small $Coef_{i}$ value. The coefficient increases during the vegetative growth period, and reaches its maximum during the reproductive growth period, following an “S”-shaped curve pattern (Shaw and Meyer, 2015). Therefore, to simulate this characteristic using the sigmoid function, the formula is as follows (Hu, 2023):


(14) $$Coef_{i}=\frac{1}{1+e^{\left(-0.06*i+5.0\right)}}\;$$

**Figure 2:**
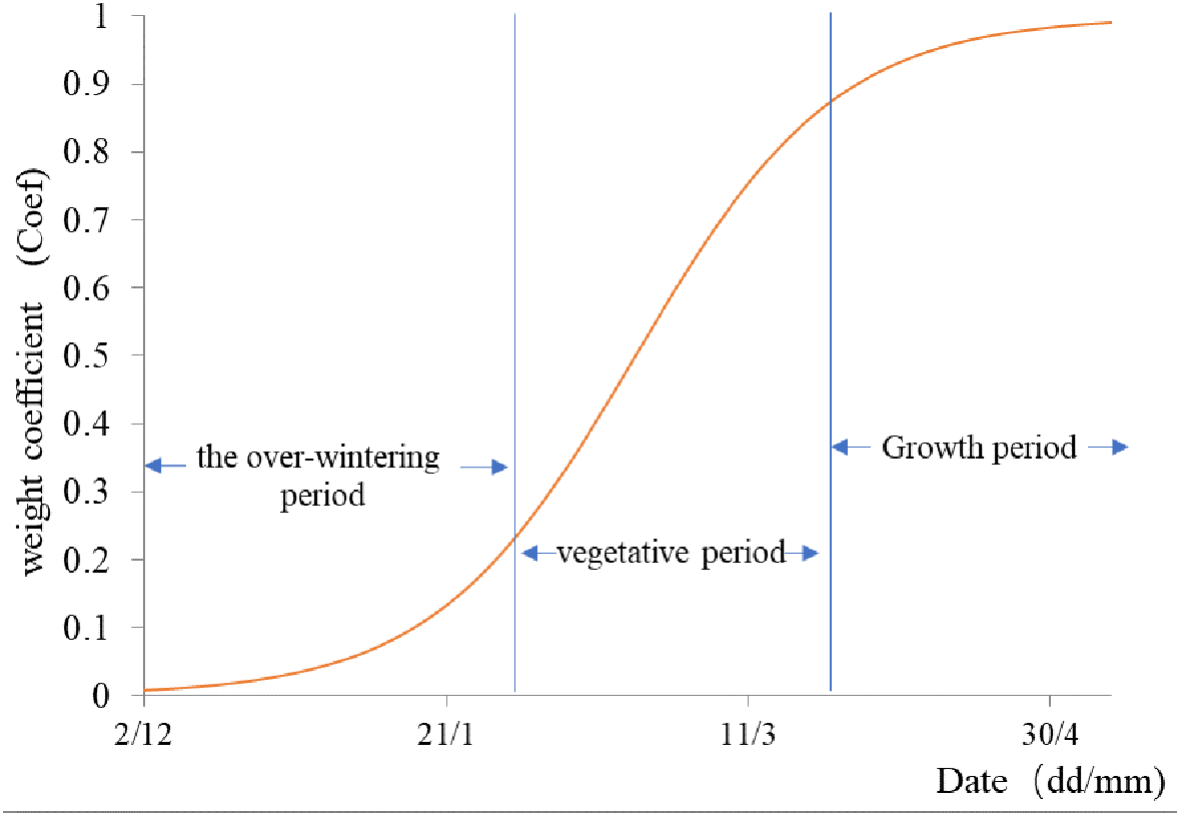
The weighting coefficient (Coef) for winter wheat waterlogging damage.

Where *i* represents the number of days from November 30th of the previous year. During the period from winter wheat sowing to November 30th, winter wheat is in its seedling stage, and it is assumed that waterlogging has no impact on its growth, therefore the $Coef_{i}\;$ value is 0. The curve showing the variation of $Coef_{i}$ with date can be obtained by calculating according to equation (14), as shown in **Figure 2**.

#### 2.3.4 New waterlogging injured index (*WI_Plus_*)

We modify the value of CritWFPS in Eq. (1), keeping the other processes unchanged, and calculate the new waterlogging injured index (*WI_Plus_*).

The format of the relative increase ratio D_soil_ of root respiration at soil temperature T_soil_ (°C) is following (Amthor, 2000):


(15) $${\text{D}}_{\text{soil}}={\text{D}}_{10}^{({\text{T}}_{\text{soil}}-10/10)}$$

D_10_ is the relative increase ratio at a temperature increase of 10°C, equals 2.0 (Lloyd and Taylor, 1994).

According to the above formula, the critical value of the water filled pore space ( CritWFPS ) at which CritWFPS = 0.65 can be modified as follows:


(16) $$CritWFPS_{\text{soil}}=CritWFPS*\;(0.8+0.2/D_{soil})$$

After replacing CritWFPS in equation (1) with $CritWFPS_{\text{soil}}$ and combining it with Eq. (12) to calculate $WI_{\;Plus}$ .

## 3 Results and analysis

### 3.1 The impact of waterlogging duration on the rate of increase or decrease in wheat yield

**Figure 3:**
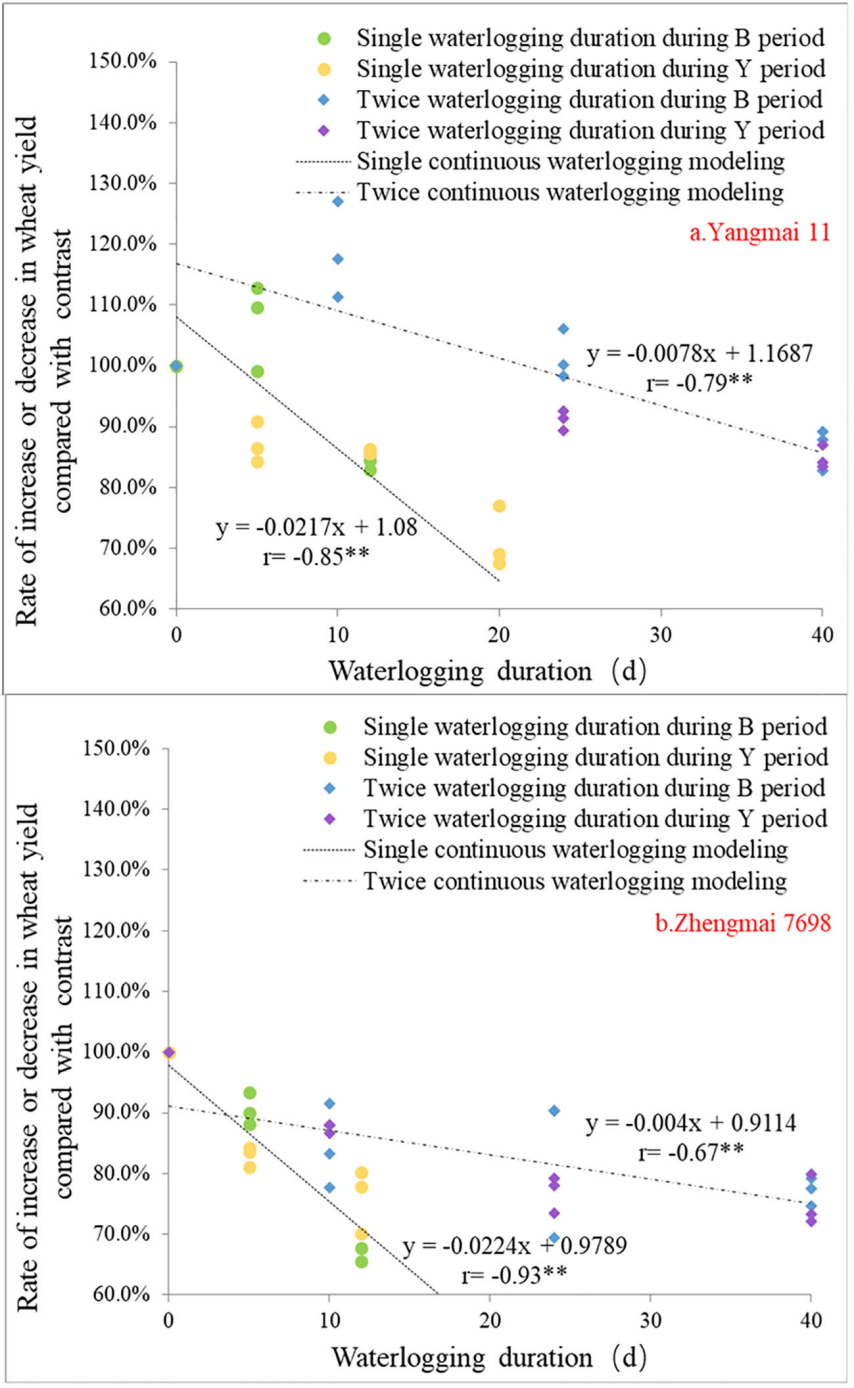
The relationship between the length of waterlogging and the rate of increase or decrease in wheat yield under different waterlogging treatments [ “a.Yang mai 11” and “b.Zheng mai 7698”]. **represented extremely significant correlation (*P*<0.01).

The study analyzed the impact of single or double continuous waterlogging during two different growth stages (jointing stage to heading stage, B period; or heading stage to maturity stage, Y period) on the yield of two wheat varieties, “Yang mai 11” and “Zheng mai 7698”. The duration of waterlogging was calculated using formula (3), and the relationship between waterlogging duration and wheat yield rate is shown in **Figure 3**. It can be observed from the figure that there is a highly significant correlation (p<0.01) between the number of days of waterlogging and the wheat yield rate. The correlation coefficients were -0.85 (“Yang mai 11”, single continuous waterlogging), -0.79 (“Yang mai 11”, double continuous waterlogging), -0.93 (“Zheng mai 7698”, single continuous waterlogging), and -0.67 (“Zheng mai 7698”, double continuous waterlogging). This means that the longer the duration of waterlogging, the lower the wheat yield rate.

The linear simulation curves of the rate of increase or decrease in yield in relation to the duration of waterlogging show significant differences between single continuous waterlogging and double continuous waterlogging treatments. The slope of the curve for the single continuous waterlogging treatment is greater than that of the double continuous waterlogging treatment, indicating that the rate of yield increase/decrease of wheat is also influenced by the number of waterlogging events. When the duration of waterlogging is the same, the rate of yield increase/decrease of wheat during the Y period is significantly lower than that during the B period. The impact of single continuous waterlogging treatment on the rate of increase or decrease in wheat yield during the Y period is more pronounced for both “Yang mai 11” and “Zheng mai 7698” varieties compared to the double continuous waterlogging treatment.

Although there is a highly significant negative linear correlation between the duration of waterlogging and the rate of increase or decrease in wheat yield, using only the duration of waterlogging is not sufficient to represent the extent of crop waterlogging. The parameters of the linear simulation curve are evidently related to the duration of continuous waterlogging, the number of waterlogging events, and the varieties affected by waterlogging.

### 3.2 The relationship between five WI indices and wheat yield

**Figure 4:**
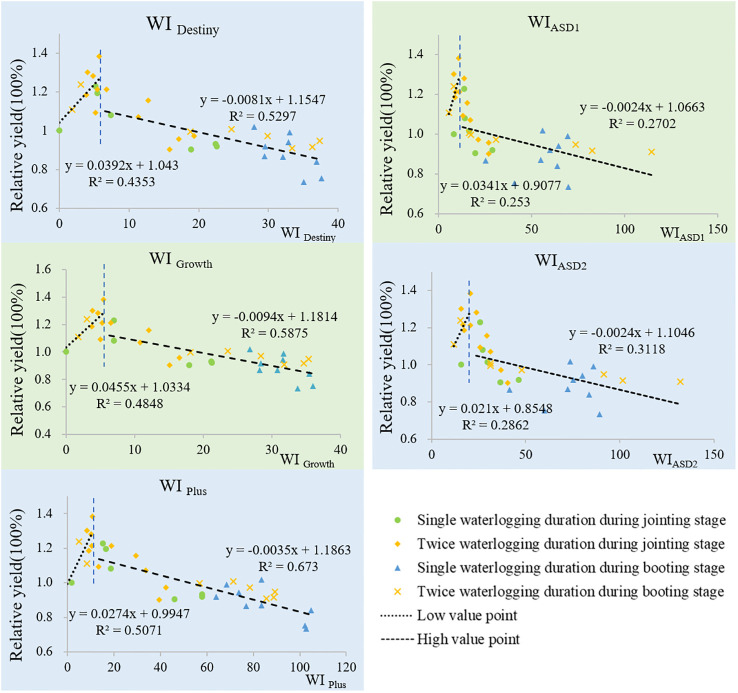
Fitted curves of five WI indices with the relative yields of “Yang mai 11”.

**Figure 5:**
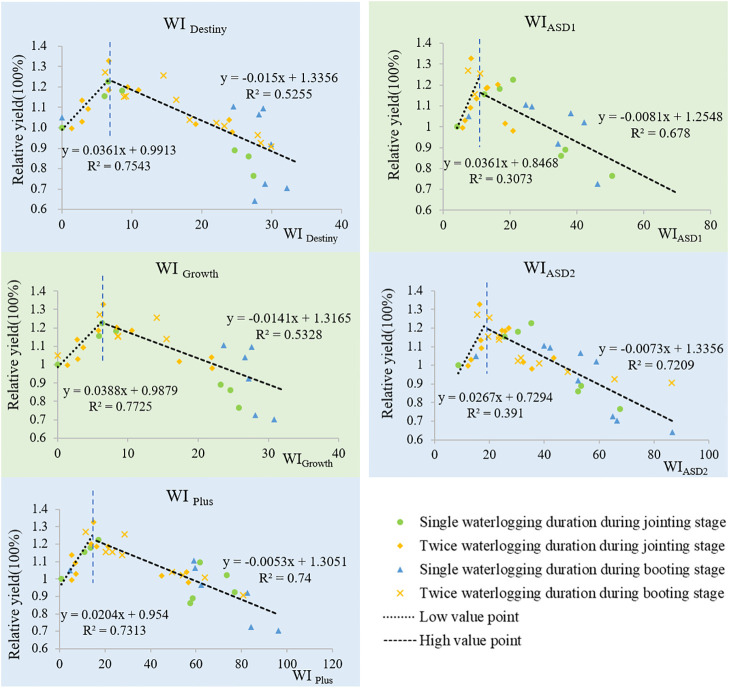
Fitted curves of five WI indices with the relative yields of “Zheng mai 7698”.

Under all treatments,the relationship between the five Wl indices and the relative yield of two wheat varieties is shown in **Figure 4** (“Yang mai 11”) and **Figure 5** (“Zheng mai 7698”). From the graph, we can observe that the fitted curves between the WI indices and relative yield of wheat exhibit a clear upward trend initially, reaching a certain point before showing a downward trend. We refer to the ascending stage as the first stage and the descending stage as the second stage, with the WI value at the inflection point referred to as the threshold.

The fitted curves of the five WI indices with wheat yield can reflect the tolerance of wheat varieties to waterlogging. The variety “Yang mai 11” exhibits strong tolerance to waterlogging, while “Zheng mai 7698” is not tolerant. In the second stage, the fitted curves between the five WI indices and the yield of “Zheng mai 7698” show a steeper slope compared to the fitted curves between the same indices and the yield of “Yang mai 11”. This means that under the same intensity of waterlogging stress, the decrease in yield for “Zheng mai 7698” is significantly greater than that for “Yang mai 11”.

(1) In the First Stage

From **Table 1**, it can be seen that in both wheat varieties, the five indices show a positive correlation with the relative yield of wheat, indicating that larger WI values correspond to higher yields. The order of correlation coefficients is as follows: 0.71> 0.70> 0.66> 0.53> 0.50, which corresponds to WI _Plus_ > WI _Growth_> WI _Destiny_> WI_ASD2_> WI_ASD1_ (“Yang mai11”); 0.88> 0.87> 0.86> 0.63> 0.55, which corresponds to WI _Growth_>WI _Destiny_ >WI _Plus_ >WI_ASD2_>WI_ASD1_(“Zheng mai 7698”). Additionally, the results of fitting WI Growth, WI Destiny, and WI Plus with the yield of “Zheng mai 7698” all reach a highly significant level (p< 0.01), with respective ‘r’ values of 0.88, 0.87, and 0.86. WI Growth, WI Plus, and WI Destiny show significant fits with the yield of “Yang mai 11” (p< 0.05) with respective ‘r’ values of 0.70, 0.66, and 0.66.

**Table 1:** The simulation of the five WI and the relative yields of Wheat in the First Stage.

variety	Five Waterlogging Index	Simulation curves	*R*²	*r*
Yang mai 11	WI _Plus_	y=0.0274x+0.9947	0.51	0.71*
	WI _Growth_	y=0.0455x+1.0334	0.48	0.70*
	WI _Destiny_	y=0.0392x+1.043	0.44	0.66*
	WI _ASD2_	y=0.021x+0.8548	0.29	0.53
	WI _ASD1_	y=0.0341x+0.9077	0.25	0.50
Zheng mai 7698	WI _Growth_	y=0.0388x+0.9879	0.77	0.88**
	WI _Destiny_	y=0.0361x+0.9913	0.75	0.87**
	WI _Plus_	y=0.0204x+0.954	0.73	0.86**
	WI _ASD2_	y=0.0267x+0.7294	0.39	0.63
	WI _ASD1_	y=0.0361x+0.8468	0.31	0.55

**represented extremely significant correlation (P<0.01). *represented significant correlation (P<0.05). The same as follow.

In the first stage, the effect of the three indexes of WI _Plus_, WI _Growth_ and WI _Destiny_ were better than that of WI_ASD1_ and WI_ASD2_.

(2) In the Second Stage

According to **Table 2**, the fitting results of the five WI indices with the relative yield of both wheat varieties reach a highly significant level of 0.01.

**Table 2:** The simulation of the five WI and the relative yields of Wheat in the Second Stage.

variety	Five Waterlogging Index	Simulation curves	*R*²	*r*
Yang mai 11	WI _Plus_	y=-0.0035x+1.1863	0.67	-0.82**
	WI _Growth_	y=-0.0094x+1.1814	0.59	-0.77**
	WI _Destiny_	y=-0.0081x+1.1547	0.53	-0.73**
	WI _ASD2_	y=-0.0024x+1.1046	0.31	-0.56**
	WI _ASD1_	y=-0.0024x+1.0663	0.27	-0.52**
Zheng mai 7698	WI _Plus_	y=-0.0053x+1.3051	0.74	-0.86**
	WI _ASD2_	y=-0.0073x+1.3356	0.72	-0.85**
	WI _ASD1_	y=-0.0081x+1.2543	0.68	-0.82**
	WI _Growth_	y=-0.0141x+1.3165	0.53	-0.73**
	WI _Destiny_	y=-0.015x+1.3356	0.52	-0.72**

**represented extremely significant correlation(P<0.01). *represented significant correlation(P<0.05). The same as follow.

The five indices show a negative correlation with the relative yield of both wheat varieties, indicating that larger WI values are associated with more severe waterlogging and lower wheat yields. The order of the absolute correlation coefficients (|r|) is as follows: 0.82 > 0.77 > 0.73 > 0.56 > 0.52, corresponding to WI _Plus_ > WI _Growth_ > WI _Destiny_ >WI_ASD2_>WI_ASD1_ (“Yang mai 11”); 0.86 > 0.85 > 0.82 > 0.73 > 0.72, corresponding to WI _Plus_ >WI_ASD2_>WI_ASD1_> WI _Growth_ > WI _Destiny_ (“Zheng mai 7698”). WI _Plus_ reflects the waterlogging severity of wheat better, and it has the highest R and |r| values, regardless of whether it is for “Zheng mai 7698” or “Yang mai 11”.

WI_ASD1_ and WI_ASD2_ show a good correlation with the yield curve of “Zheng mai 7698”, second only to WI _Plus_, while their correlation with the yield curve of “Yang mai 11” is the lowest. Upon comparison, it is found that the order of correlation between WI _Plus_, WI _Growth_ and WI _Destiny_ with the yield curve of both varieties is WI _Plus_ > WI _Growth_ > WI _Destiny_.

In conclusion, the use of low oxygen stress as the waterlogging index (WI) can quantitatively characterize the degree of waterlogging in winter wheat. The improved WI _Plus_, which takes into account soil temperature and growth stage, better reflects the waterlogging severity of wheat.

### 3.3 Validation of the accuracy of the improved index (WI _Plus_ and WI _Growth_)

**Figure 6:**
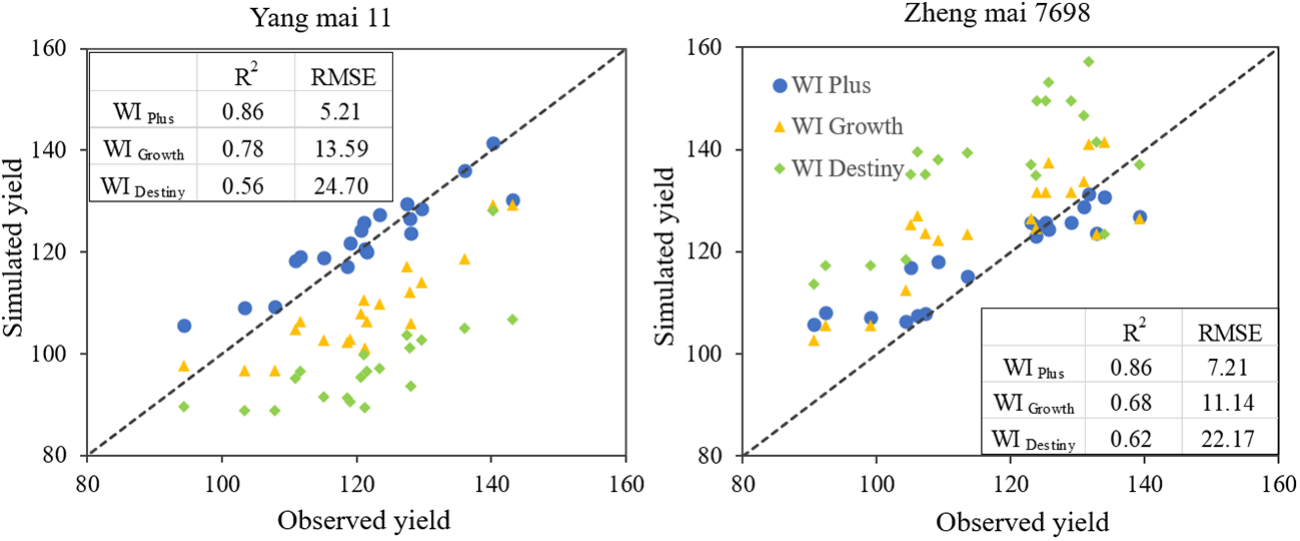
Comparison of observed (Observed) and simulated (Sim). The left is “Yang mai 11”, and the right is “Zheng mai 7698”. R^2^ represents the coefficient of determination, and RMSE represents the Root Mean Squared Error.

We used the experimental data of soil moisture, temperature, and growth period in 2021 to calculate the values of WI _Destiny_, WI _Growth_, and WI _Plus_ for 2021. Then we calculated the predicted yields by bringing the three WI values into the equations in **Tables 1**, **2**. Compared with the observed yield, it was found that the improved WI _Plus_ index provided more accurate predictions for the yield of two wheat varieties (**Figure 6**).

## 4 Discussion

The middle and lower reaches of the Yangtze River region are the main wheat-growing areas in China. They are greatly influenced by the subtropical monsoon climate. In the months of March and April each year, extensive waterlogging occurs, and water stress can lead to a decrease in wheat yield or even complete crop failure (Ghobadi et al., 2017). This study has established five WI indices and analyzed them through pot experiments on two wheat varieties, “Yang mai 11” and “Zheng mai 7698,” under different waterlogging treatments.

Among the four waterlogging treatments, there is a strong negative correlation between the duration of waterlogging and the wheat yield increment/decrement rate. In other words, the longer the waterlogging period, the lower the wheat yield increment/decrement rate. When comparing the effects of waterlogging during the jointing stage and the booting stage on wheat yield increment/decrement rate, it was found that waterlogging during the booting stage had a greater impact on wheat yield compared to the jointing stage, which is consistent with the findings of Ghobadi et al. Additionally, the study discovered that, under the same duration of waterlogging, the slope of the yield increment/decrement curve for a single waterlogging treatment was greater than that of the curve for two waterlogging treatments. This could be attributed to the adaptive phase that occurs when the waterlogging duration reaches a critical point (5 days) in wheat growth, where the crop gradually adapts and recovers normal growth. This includes processes such as stomatal regulation, physiological metabolism adjustment, root adaptation, and nutrient regulation to cope with the impact of waterlogged conditions. During this phase, wheat relies on self-regulation and adaptive capacity to withstand the stress caused by waterlogging and maintain growth and yield (Shaw and Meyer, 2015). Therefore, although there is a strong negative correlation between the duration of waterlogging and the wheat yield increment/decrement rate, the linear simulation curve parameters of the waterlogging duration and wheat yield increment/decrement rate are significantly influenced by factors such as the waterlogging period, frequency, and wheat variety. Using only the duration of waterlogging as a universal characteristic to represent the degree of waterlogging in wheat is not highly applicable.

It has been found through research that there is a correlation between the five WI indices and wheat yield. Before reaching the threshold, the fitting curve of the five WI indices and wheat yield shows an upward trend, indicating that a higher WI value corresponds to a higher wheat yield. However, once the threshold is exceeded, the curve starts to decline, and an increase in WI value will lead to a decrease in wheat yield. In the second stage of the fitting curve, the five WI indices show the following characteristics in relation to wheat yield: for the “Zheng mai 7698” variety, the curve’s slope relating to the WI index and yield is greater than that of the “Yang mai 11” variety. This indicates that the “Yang mai 11” variety has stronger tolerance to waterlogging, while the “Zheng mai 7698” variety is less capable of withstanding waterlogged conditions, which is consistent with the findings of Yu J J and others (Yu et al., 2014). Therefore, it can be seen that the WI indices are influenced by the tolerance of the variety to waterlogging, although the present study did not consider the tolerance of different varieties. However, the WI indices can reflect the level of waterlogging tolerance among different wheat varieties.

Many studies have shown a positive correlation between soil respiration and temperature (Lloyd and Taylor, 1994; Das and Paul, 2015). Considering the influence of temperature on soil oxygen content under waterlogged conditions where oxygen diffusion is restricted, the commonly used fixed oxygen threshold may not be suitable. Experimental results on wheat also indicate that the improved WI _Plus_ is more suitable for quantitatively analyzing the impact of low oxygen stress on wheat yield. The threshold for WI _Plus_ is 14.9, where WI _Plus_ ≤ 14.9 (“Zheng mai 7698”) and ≤ 10.8 (“Yang mai 11”) show a positive correlation between WI _Plus_ and wheat yield, indicating that the wheat is not waterlogged. When WI _Plus_ > 14.9 (“Zheng mai 7698”) and > 10.8 (“Yang mai 11”), there is a negative correlation between WI _Plus_ and wheat yield, indicating that wheat is affected by waterlogging, and a higher WI value indicates more severe waterlogging.

This study calculates the soil aeration index based on soil volumetric water content and constructs the WI index using characteristic values of wheat root responses to low oxygen (anoxic) stress, making it applicable in general. Additionally, advancements in remote sensing technology, particularly satellite remote sensing, have shown that soil moisture can be measured using various remote sensing techniques (Wang et al., 2022). Firstly, spectral pictures of wheat planting regions may be collected using high-resolution remote sensing equipment carried by satellites or drones. Secondly, we can preprocess the collected images, including radiation calibration, atmospheric correction, geocoding, and other steps, to eliminate noise and distortion in the images and ensure the accuracy of the data. Thirdly, using image processing technology, extract features related to wheat waterlogging, such as soil temperature and soil moisture content. Finally, the relevant feature quantities were brought into the exponential model of this study, and evaluated and verified through yield data. This can achieve a wider range of wheat stain monitoring. However, its practical application is limited by factors such as insufficient temporal resolution, difficulty in extracting characteristic quantities of waterlogging, and large computational load. Therefore, in the application process, these factors need to be fully considered, and these effects can be reduced through optimization methods.

Currently, there are three remote sensing methods commonly used for soil moisture retrieval: optical remote sensing, thermal infrared remote sensing, and microwave remote sensing. Among them, microwave remote sensing is unaffected by various meteorological conditions and has strong cloud penetration capability. Radar backscatter coefficients are sensitive to changes in soil moisture (Li et al., 2020; Wang et al., 2022). For example, Xiong Q X., et al. used Sentinel-1A data to extract soil surface relative moisture content data. Then, combined with the precipitation index in the previous period, they used the Kalman filter interpolation method to obtain daily soil moisture information in the observation area. In addition, remote sensing technology can also obtain soil temperature information (Xiong et al., 2021). This provides more opportunities for the widespread application of the WI index in monitoring waterlogging. However, since this experiment was conducted in pots, there is minimal vertical variation in soil volumetric water content, which significantly differs from field moisture characteristics. If the results are further applied in field conditions, it would be an area for future improvement.

## 5 Conclusions

Using only the duration of waterlogging cannot quantitatively reflect the severity of wheat waterlogging. It is also related to factors such as the tolerance of wheat varieties and the frequency of waterlogging events. Furthermore, the WI index can be applied to assess waterlogging damage under excessive irrigation conditions and can also reflect the impact of multiple waterlogging events on wheat yield. Comparing WI _Destiny_, WI _Growth_, WI _ASD1_ and WI _ASD2_, the improved WI _plus_ shows better performance. When WI _plus_ is ≤14.9 (“Zheng mai 7698”) or ≤10.8 (“Yang mai 11”), wheat does not experience waterlogging. When WI _plus_ is >14.9 (“Zheng mai 7698”) or >10.8 (“Yang mai 11”), wheat experiences waterlogging.

## Data availability statement

The original contributions presented in the study are included in the article/**Supplementary Material**. Further inquiries can be directed to the corresponding author.

## Author contributions

F-LX: Writing-original draft, Formal analysis, Methodology. P-MH: Writing – review & editing, Funding acquisition. XW: Writing – review & editing, Date curation, Investigation. MH: Writing – review & editing, Revise the paper. KL: Writing – review & editing, Revise the paper. Q-XX: Writing – review & editing, Conceptualization, Methodology. All authors contributed to the article and approved the submitted version.
